# Scotch Asylums

**Published:** 1888-11-03

**Authors:** 


### SCOTCH ASYLUMS.

### REPORT OF THE COMMISSIONERS IN LUNACY FOR SCOTLAND.

Alienist physicians, whether on this or on the northern side of the border, always look anxiously for the Blue book of the Scotch Commissioners; experience having told them that it is a model of conciseness and completeness. The present number is no exception to the rule. Everything is mentioned, and yet nothing seems to have undue prominence given to it. We should call it a succinct history of the insane population of Scotland for the past year.

Eleven thousand six hundred and nine insane persons were known to exist in Scotland at the close of 1887. Of these 9,760 were paupers, 1,797 were private patients, and 52 were maintained at the expense of the state—that is they were criminal lunatics. Thirty years since the paupers numbered only 4,737, and the private cases only 1,032. These numbers show, among other things, that the pauper lunatics have increased in a higher ratio than the private. In Scotland the whole of the pauper lunatics who are in asylums at all are in public institutions, a state of affairs which ought to pertain in England also, and which it is to be hoped the new Lunacy Bill will insist on. Another striking point in the statistics is that the vast majority of the private patients are in public institutions. In fact there are only 148 in private asylums, and five private asylums suffice for the whole of Scotland.

Not fewer than 42 cases are voluntarily in the asylums, that is they are not under the usual certificates, and may leave at any moment thay please.

In the royal and district asylums, the percentage of recoveries calculated on the admissions was 40, while in private asylums it was only 27, from which it may be inferred that the curable cases seek the benefits of public institutions rather than private ones. In public asylums the death-rate was 7·9 per cent on the numbers resident, and in private asylums it was 5·1 per cent.

Much of the welfare of the patients depends on obtaining and retaining the services of suitable attendants, and it is too true that scarcely any inducement is sufficiently strong to ensure these. There were 458 changes among the attendants in Scotch asylums during the year, and it is interesting to note some of the causes of these changes. Ninety-seven men and 215 women left voluntarily, three women absconded, nine men were dismissed for drunkenness, six men and two women for insubordination, four men and five women for being absent without leave, two men and 19 women for incompetence, six men and three women for carelessness, and eight men and seven women for complaints of patients.

Two hundred and twenty-one patients escaped, bein at the rate of 25 per 1,000 of the number resident, a proportion which to us seems extremely high, but it need not be altogether deplored provided it may be construed to mean a great amount of personal freedom enjoyed by the inmates of Scotch asylums generally. The same remark may be made of the suicides, which were seven in number, being at the rate of about one case in 1,600, whereas in England it was only one in about 4,300.

The practice of boarding-out pauper lunatics in private houses is carried on to a great extent, not fewer than 2,270 being so cared for at the date this report, and there were also 132 private patients residing in private families.

Judging from a statement in the report it would seem difficult to obtain accommodation for the class just above paupers, and yet upwards of 1,000 private patients are kept at rates below a guinea a-week.

The total cost of maintenance for paupers has risen from £80,000 per annum in 1858 to £225,000 in 1887, and the cost per head has increased from £20 to £25 per annum. In England the cost of maintenance per head has been going steadily down, and it is not easy to see why it should rise in Scotland.

Under the heading "Alien Lunatics," we learn that 29 pauper lunatics were removed from Scotland as they had no legal settlement in that country. Ten of these were sent to England, and 19 to Ireland. It is a complete mystery to those having to deal with pauper lunatics in England why a similar law cannot be made applicable here. There are some thousands of Irish and Scotch pauper lunatics in English asylums maintained at the expense of the English ratepayers, and this expense must far exceed £50,000 a-year, and yet nothing is done. Verily John Bull is a longsuffering animal. They manage these things better in Scotland.

The Appendix to the Report contains the entries, left in the visitors' books at the various public and private asylums and workhouses. There is also a most instructive sketch of the condition of the insane in private dwellings.

